# Effects of stress ulcer prophylaxis in adult ICU patients receiving renal replacement therapy (Sup-Icu RENal, SIREN): Study protocol for a pre-planned observational study

**DOI:** 10.1186/s13063-017-2408-3

**Published:** 2018-01-10

**Authors:** Joerg C. Schefold, Anders Perner, Theis Lange, Jørn Wetterslev, Matt P. Wise, Mark Borthwick, Stepani Bendel, Frederik Keus, Anne Berit Guttormsen, Søren Marker, Mette Krag, Morten Hylander Møller, for the SUP-ICU investigators

**Affiliations:** 1 Department of Intensive Care Medicine, Inselspital, Bern University Hospital, University of Bern, Freiburgstrasse 18, CH 3010 Bern, Switzerland; 2 Department of Intensive Care 4131, Copenhagen University Hospital, Rigshospitalet, Copenhagen, Denmark; 3 Centre for Research in Intensive Care, Copenhagen University Hospital, Rigshospitalet, Copenhagen, Denmark; 4 Section of Biostatistics, University of Copenhagen, Copenhagen, Denmark; 5 Copenhagen Trial Unit, Centre for Clinical Intervention Research, Copenhagen University Hospital Rigshospitalet, Copenhagen, Denmark; 6 Department of Adult Critical Care, University Hospital of Wales, Cardiff, UK; 7 Pharmacy Department, Oxford University Hospitals NHS Trust, Oxford, UK; 8 Department of Intensive Care Medicine, Kuopio University Hospital, Kuopio, Finland; 9 Department of Critical Care, University Medical Center Groningen, University of Groningen, Groningen, The Netherlands; 10 Department of Anaesthesia and Intensive Care, Haukeland University Hospital and Clinical Institute 1 UiB, Bergen, Norway; 11 Department of Intensive Care 4131, Copenhagen University Hospital, Rigshospitalet, Blegdamsvej 9, Copenhagen, 2100 Denmark

**Keywords:** Dialysis, Acute kidney injury, Acute kidney injury, Acute renal failure, Pantoprazole, Sepsis

## Abstract

**Background:**

Proton pump inhibitors are often used in critically ill patients to prevent gastrointestinal bleeding despite limited evidence for benefit. Patients with acute kidney injury requiring renal replacement therapy (RRT) are at high risk of gastrointestinal bleeding as (pre-)uremia induces coagulopathy through effects on platelets and coagulation cascades. No high-quality randomized clinical trials have previously assessed the benefits and harms of prophylactic proton pump inhibitor use in this high-risk population of adult critically ill patients.

**Methods/design:**

Among the 3350 patients included in the Stress Ulcer Prophylaxis in the Intensive Care Unit (SUP-ICU) trial—an investigator-initiated international randomized clinical trial on prophylactic proton pump inhibitor versus placebo in acutely admitted adult ICU patients at risk of gastrointestinal bleeding—we will compare the benefits and harms of prophylactic use of proton pump inhibitor in patients in need of RRT versus those not requiring this treatment. We will determine the proportion of patients with clinically important bleeding, the proportion of patients with adverse events including pneumonia, *Clostridium difficile* enteritis, or acute myocardial ischemia in the ICU, as well as transfusion requirements. Moreover, 90 day and 365 day mortality post-randomization will be investigated. As a secondary analysis, we will examine the association between acute kidney injury and RRT during ICU stay and gastrointestinal bleeding.

**Discussion:**

With the outlined predefined analysis, we will characterize the balance between the benefits and harms of stress ulcer prophylaxis in acutely admitted adult ICU patients in need of RRT, including the potential interaction of allocation to proton pump inhibitor versus placebo.

**Trial registration:**

ClinicalTrials.gov, NCT02718261. Registered on 14 March 2016.

**Electronic supplementary material:**

The online version of this article (doi:10.1186/s13063-017-2408-3) contains supplementary material, which is available to authorized users.

## Background

In a general population of mixed adult intensive care unit (ICU) patients, overt gastrointestinal bleeding episodes present in about 5% of cases [1]. Clinically important gastrointestinal bleeding episodes, which may impact patient’s morbidity and mortality, occur in about 3% of cases [1, 2]. In critically ill patients, it is assumed that bleeding episodes may at least partly result from stress-induced gastric mucosal damage, and guidelines recommend stress ulcer prophylaxis with proton pump inhibitors or histamine-2-receptor antagonists [3]. However, the evidence for prophylactic stress ulcer prophylaxis to prevent gastrointestinal bleeding in critically ill patients is low in quantity and quality [4].

In the population of ICU patients with RRT-dependent acute kidney injury, the rate of gastrointestinal bleeding episodes may be higher, owing to effects of uremia on platelets and coagulation cascades [5–10]. Moreover, renal replacement therapy (RRT) activates coagulation pathways, owing to contact with foreign material, and requirement for anticoagulation of the extracorporeal circuit may further impair coagulation [6–15]. Although acute kidney injury is frequently observed in critically ill patients and negatively impacts outcome [5, 12, 16, 17], only a few previous prospective studies have reported on the incidence and severity of gastrointestinal bleeding in this population. Recent data from a large randomized clinical trial comparing RRT modalities in critically ill patients [18] indicated that clinically relevant all-cause bleeding episodes may be observed in up to 30% of patients receiving RRT [8, 19]. However, the trial was conducted in the era prior to the widespread use of regional RRT anticoagulation (e.g. using citrate), and only all-cause bleeding was assessed. Accordingly, the collection of contemporary data and data on gastrointestinal bleeding in critically ill patients requiring RRT is warranted. Moreover, although the use of prophylactic proton pump inhibitors in critically ill patients requiring RRT may reduce the risk of gastrointestinal bleeding, mounting data suggest an increased risk of serious adverse events, including increased risk of nosocomial infections (e.g. hospital-acquired pneumonia and *Clostridium difficile* enteritis) and adverse cardiovascular events [4, 20].

As for the general ICU population, there are no data from high-quality randomized clinical trials on the benefits and harms of prophylactic proton pump inhibitors in RRT-treated critically ill patients. Of note, the use of proton pump inhibitors in critically ill patients, including RRT-treated patients, is considered off-label use. Consequently, we aim to assess the balance between the benefits and harms of prophylactic use of proton pump inhibitors in adult ICU patients with acute kidney injury requiring RRT. We hypothesize that proton pump inhibitor use reduces the risk of gastrointestinal bleeding, but increases the risk of nosocomial infections.

### Objectives

The primary objective of SIREN is to compare the rate of gastrointestinal bleeding episodes in critically ill patients with or without need for RRT. Secondary objectives of SIREN are to balance the benefits and harms of stress ulcer prophylaxis in this patient population.

## Methods

**Fig. 1:**
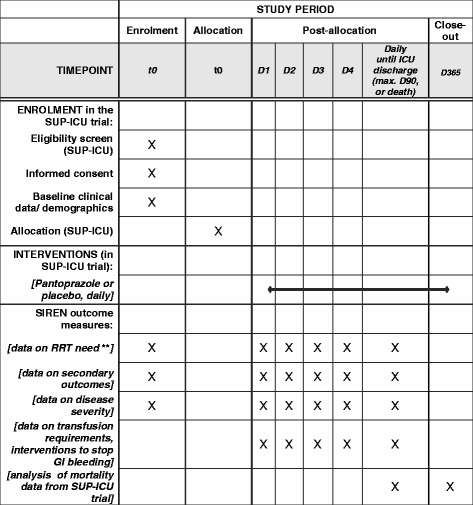
SIREN (pre-planned sub-analysis of the SUP-ICU trial) data * * Detailed information on SUP-ICU trial assessments is provided in [22]. ** RRT = Renal replacement therapy

Sup-Icu RENal (SIREN) is a pre-planned sub-analysis (NCT02718261) of the Stress Ulcer Prophylaxis in the Intensive Care Unit (SUP-ICU) trial (protocol no. 3.0, dated 20 October 2015) [21, 22]. This manuscript was prepared according to the Strengthening the Reporting of Observational Studies in Epidemiology (STROBE) statement [23]. A STROBE checklist (Additional file 1) and a SPIRIT checklist (Additional file 2 and Fig. 1) are attached.

### SUP-ICU trial

The SUP-ICU trial is an investigator-initiated, pragmatic, international, multicenter, randomized, blinded, parallel-group trial where 3350 acutely admitted adult ICU patients with one or more risk factors for gastrointestinal bleeding are randomized to stress ulcer prophylaxis with 40 mg pantoprazole (Actavis, Gentofte, Denmark) or placebo intravenously once daily during their ICU stay (given until ICU discharge or death, for a maximum of 90 days). The SUP-ICU trial medication will be blinded to the patient, the investigators, the outcome assessors, the data manager, and the statistician conduction the analysis. An independent company (NOMECO Clinical Trial Supply Management, CTSM, Copenhagen, Denmark) will handle masking, coding, and distribution of trial medication. Trial site staff will perform computer-based (central) randomization and trial data will be collected via electronic case report files. Details of the trial have been published elsewhere [22]. Some 33 ICUs in six European countries (Denmark, Norway, Finland, Switzerland, the Netherlands, and the UK) are randomizing patients [22].

### Approvals

The SUP-ICU trial is approved by the Danish Health and Medicine Agency (2015030166), the Committees on Health Research Ethics in the Capital Region of Denmark (H-15003141), the Danish Data Protection Agency (RH-2015-3203695), and by all respective national regulatory bodies in the participating countries, and was registered at clinicaltrials.gov (no. NCT02467621) [22].

### Population

The SUP-ICU trial [22] will include critically ill adult patients acutely admitted to one of the participating ICUs with one or more of the following risk factors for gastrointestinal bleeding:

1. Shock (continuous infusion of vasopressors or inotropes, systolic blood pressure < 90 mmHg, mean arterial blood pressure < 70 mmHg *or* plasma lactate level of ≥ 4 mmol/l)
2. Need for intermittent or continuous RRT
3. Use of invasive mechanical ventilation expected to last more than 24 hours
4. Acute (within previous 24 hours) coagulopathy or history of coagulopathy (platelet count < 50 × 10^9^/l *or* international normalized ratio > 1.5 *or* prothrombin time > 20 s within the 6 months prior to hospital admission)
5. Ongoing treatment with any anticoagulant (prophylactic doses excluded)
6. History of chronic liver disease (portal hypertension, cirrhosis proven by biopsy, computed tomography scan, or ultrasound *or* history of variceal bleeding or hepatic encephalopathy)

Exclusion criteria include contraindications to proton pump inhibitors, current daily use of any proton pump inhibitor or any histamine-2-receptor antagonist, gastrointestinal bleeding of any origin during current hospital admission, diagnosis of peptic ulcer during current hospital admission, organ transplant during current hospital admission, withdrawal from active therapy or brain death, fertile woman with positive test for urinary or plasma human chorionic gonadotropin, and patients in whom consent according to national regulations cannot be obtained [22]. In SIREN the population of interest is patients with acute kidney injury receiving RRT.

### Exposure

A) RRT within 3 days: All patients receiving RRT at time of randomization, and patients needing RRT within the first 72 hours following randomization.
B) RRT after 3 days: All patients requiring RRT after 72 hours following randomization.

### Comparator

No RRT: All patients without need of RRT at time of randomization. If a patient subsequently receives RRT she or he will be moved from the “no RRT” group to the “RRT within 3 days” group if the first RRT session occurs within 72 hours and the “RRT after 3 days” group if the first RRT session occurs after 72 hours.

### Outcome measures in SIREN

#### Primary outcome measure

Proportion of patients with clinically important gastrointestinal bleeding (as defined in the next section).

#### Secondary outcome measures

1. Proportion of patients with one or more of the following adverse events: clinically important gastrointestinal bleeding, pneumonia, *C. difficile* infection, or acute myocardial ischemia in the ICU
2. Proportion of patients with serious adverse reactions
3. Proportion of patients with one or more infectious adverse events (pneumonia or *C. difficile* infection) in the ICU
4. Days alive without use of mechanical ventilation, RRT, or circulatory support in the 90 day period
5. 90 day mortality post-randomization (if available, 1 year mortality, i.e. 365 days)
6. Proportion of patients receiving treatment (interventions) to stop gastrointestinal bleeding (i.e. endoscopy, open or laparoscopic surgery, or coiling)
7. Number of units of packed red blood cells transfused

### Definitions

#### Clinically important gastrointestinal bleeding

Clinical observation of hematemesis, coffee ground emesis, melena, hematochezia, or bloody nasogastric aspirate *and* fulfillment of one or more of the following criteria:

- Spontaneous drop in systolic blood pressure, mean arterial pressure or diastolic pressure of 20 mmHg or more within 24 hours after the gastrointestinal bleeding episode, in the absence of other causes
- Vasopressor (i.e. norepinephrine, epinephrine, dopamine, vasopressin, or terlipressin) initiated or increased by 20% or more within 24 hours after the gastrointestinal bleeding episode, in the absence of other causes
- Hemoglobin decrease by at least 2 g/dl (1.24 mmol/l) within 24 hours after the gastrointestinal bleeding episode, in the absence of other causes
- Transfusion of two or more units of packed red blood cells within 24 hours after the gastrointestinal bleeding episode, in the absence of other causes

### Data collection and management

Prospectively collected data will be entered in the SUP-ICU database via electronic case report files. Web-based access to the database is provided to researchers on a 24/7 basis. Data will be encoded by using a unique patient identification number. Data will be handled according to the requirements of local data protection authorities or respective ethical committees. All trial data will be stored in a protected environment for a minimum of 15 years and anonymized if requested by the authorities.

The following data will be collected in SIREN: age, sex, comorbidities including previous or chronic RRT, date of hospital and ICU admission, renal Sepsis-related Organ Failure Assessment (SOFA) score, total SOFA score, Simplified Acute Physiology Score (SAPS) II, date of ICU discharge, use of RRT at baseline, use of RRT within the first 3 days of ICU admission, use of RRT after 3 days following randomization, data on clinically important gastrointestinal bleeding episodes, including confirmation of bleeding and interventions to stop bleeding, number of transfused red blood cell units, use of non-steroidal anti-phlogistic or anti-inflammatory drugs at hospital admission, use of anticoagulants at hospital admission, lowest platelet count within 24 hours prior to randomization, and the outcome measures listed previously. Further details are available elsewhere [21].

### Safety

Patients can be withdrawn from the SUP-ICU trial in the case of clinical indication for use of proton pump inhibitors or histamine-2-receptor antagonists, clinical indication for withdrawal as judged by a responsible clinician or local investigator, in the case of a request for withdrawal by the patient or patient’s next of kin, and in the case of serious adverse reaction or suspected unexpected serious adverse reaction [22]. Details of serious adverse reactions and suspected unexpected serious adverse reactions are given elsewhere [22].

### Statistical analysis

SIREN data analysis will be performed by the SIREN study team after closure of the main database and according to the statistical analysis plan outlined next. We will use R (R-project.org) for the analysis.

### Missing data

In the case of missing observations of > 5% in any specific analysis, that analysis will be performed both as complete-case and using multiple imputation based on chained equations. All variables in the specific analysis will be included in the multiple imputation, as well as stratification variables (site and presence of hematological cancer), age, SOFA score at baseline, type of admission (medical, elective surgery, or emergency surgery), SAPS II score at baseline, RRT at baseline, mechanical ventilation at baseline, shock at baseline, proportion of patients with clinically important gastrointestinal bleeding, proportion of patients with one or more episodes of serious adverse events (pneumonia, *C. difficile* infection, or myocardial ischemia), and 90 day mortality. If multiple imputation is used, the primary result of the trial will be based on these data. For additional details on the handling of missing data in SUP-ICU, please refer elsewhere [21].

#### Primary analysis

The primary analysis will be a conventional subgroup analysis of the effects of pantoprazole versus placebo in patients with RRT at baseline (i.e. at randomization). We will use Cox models censoring at death or loss to follow-up and intention to treat in the two groups defined by RRT at baseline. We will include the interaction between baseline RRT and allocated treatment, which will constitute the main test of differences in effects. Statistical significance will be considered for *P* < 0.1; whether we find a statistically significant difference or not, we will report hazard ratios stratified by baseline RRT. As treatment is randomized, we will not control the analysis for any other variables than stratification variables (i.e. site and presence of hematological malignancy at randomization). Secondary outcome measures will be analyzed in a similar manner as the primary outcome measure when they can be expressed as time-to-event outcome measures (no. 4). Secondary outcome measures nos. 1–3, 5, and 6 will be analyzed using logistic regression and secondary outcome measure no. 7 will be analyzed using Poisson regression. All regressions will include the same covariates as the primary analysis.

#### Analysis of the effect of RRT

For the analysis of the effects of RRT itself, the outcome measures will be compared between the dynamically updated groups (“No RRT”, “RRT within 72 hours”, “RRT after 72 hours”) using Cox models with delayed entry. This is comparable to a Cox model with time-varying exposure, where the time-varying exposure is the dynamic group for each patient. We will adjust the analysis for the following important confounders: the investigational medicinal product (pantoprazole vs. placebo), disease severity (SAPS) II at baseline, anticoagulation at hospital admission, age, and sex. In addition, raw rates will be reported for each day of the first week of ICU admission. In the analysis of RRT itself, we will only consider time to gastrointestinal bleeding.

#### Power analysis

The sample size of SIREN is fixed. The SUP-ICU trial includes 3350 ICU patients (total cohort). We expect that 335 patients (i.e. 10%) present with acute kidney injury requiring RRT at baseline. The actual power of the primary analysis will be expressed through the width of the confidence intervals for effect parameters, which will be included in the SIREN manuscript.

### Timeline

2015: Development of research strategy and SIREN study protocol

2016–2017: Inclusion of patients to SUP-ICU

2018: SUP-ICU and SIREN data analysis, writing, and submission of manuscript

### Publication/authorships

A manuscript will be submitted to a peer-reviewed journal following publication of the SUP-ICU trial main results whether positive, negative, or neutral. Publication of SIREN will be performed concomitant to the analysis of the SUP-ICU, with a manuscript drafted within 180 days of completion of the statistical analysis. SIREN will reference the main publication as well as refer to and put respective main primary and secondary outcome measures or results into perspective. A SIREN scientific committee will be formed, which is chaired by the SIREN principal investigator (the first author of this manuscript). Authors of the SUP-ICU trial, national principal investigators, or trial site investigators will be invited as co-authors, depending on their respective personal input. The SIREN scientific committee will decide on which journal is deemed appropriate, and will grant authorships depending on personal input according to the Vancouver definitions [24].

## Discussion

### Trial rationale

The evidence is sparse [4], but use of stress ulcer prophylaxis is recommended in adult critically ill patients with risk factors for gastrointestinal bleeding, and three out of four ICU patients receive acid suppressants [3]. From a clinical perspective, critically ill patients with acute kidney injury requiring RRT constitute a high-risk group. In the outlined study, we aim to assess the balance between the benefits and harms of prophylactic proton pump inhibitors (specifically, pantoprazole) in the subgroup of patients with acute kidney injury requiring RRT. We will compare patients with and without need of baseline RRT (primary analysis), and assess the impact of developing acute kidney injury with need of RRT during ICU stay (secondary analysis). We will exclusively assess patient-important outcome measures, including clinically important gastrointestinal bleeding, nosocomial infections, and mortality. SIREN is expected to provide contemporary and important data on the risk of gastrointestinal bleeding in critically ill patients undergoing RRT, including guidance on use of acid suppressants in this population. This has important implications for patients and relatives, the health care system, and society.

### Strengths and limitations

Using data from a large international pragmatic high-quality randomized clinical trial will result in high external validity (generalizability). The extent of missing data is expected to be low, and missing data will be handled as recommended [25]. Analysis will be conducted according to the present predefined statistical analysis plan. This protocol has been prepared according to the STROBE statement [23] (Additional file 1). Results are applicable to daily clinical practice with potential direct impact on stress ulcer prophylaxis prescription practice on the ICU.

The limitations include the fixed sample size with risk of inflated estimates (type 1 error). Moreover, only the total cohort of ICU patients with need for RRT will be analyzed, and we will not be able to conclude on potential effects of specific RRT modalities (e.g. intermittent vs*.* continuous RRT), timing, or any specific mode of anticoagulation used in the context of RRT, or applied RRT dose [18, 26, 27].

In conclusion, the balance between the benefits and harms of prophylactic proton pump inhibitors in acutely ill adult ICU patients with acute kidney injury requiring RRT is unknown. SIREN aims to provide important high-quality data on this topic.

### Trial status

The SUP-ICU trial is currently actively recruiting patients. A total of 3023 patients were randomized as of August 22, 2017. Completion of patient recruitment is expected for November 2017.

## Additional files

Additional file 1: STROBE statement. (DOC 85 kb)

Additional file 2: SPIRIT 2013 checklist. (DOC 121 kb)

## Abbreviations

ICU: Intensive care unit
RRT: Renal replacement therapy
SAPS: Simplified Acute Physiology Score
SIREN: Sup-Icu RENal
SOFA: Sepsis-related Organ Failure Assessment
STROBE: Strengthening the Reporting of Observational Studies in Epidemiology
SUP-ICU: Stress Ulcer Prophylaxis in the Intensive Care Unit
